# Education and Experience as Determinants of Micro Health Insurance Enrolment

**DOI:** 10.34172/ijhpm.2020.44

**Published:** 2020-04-07

**Authors:** Basri Savitha, Subrato Banerjee

**Affiliations:** ^1^Manipal Institute of Management, Centre for Advanced Research in Financial Inclusion, Manipal Academy of Higher Education, Manipal, Karnataka, India.; ^2^University of Melbourne (Australia India Institute), Melbourne, VIC, Australia.; ^3^Queensland University of Technology (BEST Centre), Brisbane, QLD, Australia.

**Keywords:** Micro Health Insurance, India, Illness, Usurious Borrowing, Education

## Abstract

**Background:** India faces a formidable challenge of providing universal health coverage to its uninsured population in the informal sector of the economy. Numerous micro health insurance (MHI) schemes have emerged as health financing mechanisms to reduce medical-illness-induced poverty. Existing research shows that the purchase of health insurance is most likely to be determined by health status, expected healthcare expenditure, and past health experiences in addition to socio-economic variables. We add to the understanding of various factors influencing enrolment in MHI from an Indian perspective.

**Methods:** A survey was carried out to collect quantitative data in three districts in the state of Karnataka, India.

**Results:** We show that *education* does not matter as significantly as *experience* does, in the determination of new insurance purchases. In other words, the importance of new insurance is not understood by those who are merely educated, but by those who have either fallen ill, or have previously seen the hazards of usurious borrowing.

**Conclusion:** Our study provides deeper insights into the role of usurious borrowing and past illness in determining insurance purchases and highlights the formidable challenge of financial sustainability in the MHI market of India.

## Background

A considerable amount of emphasis is being put on the need to *educate* consumers on the merits of a product,^1^ “particularly in this age of rampant misinformation, a disinterested public, or the genuine possibility that customers simply don’t believe they need a given product.” The lack of a feeling of necessity (noted at the very end of the previous quote), often presents itself as a hindrance to insurance buying. For example,in a recent study,^2^ it is observed that half the respondents indicated confusion about their health insurance plans, often leading to the delay or a complete foregoing of medical care.

Therefore, targeted outreach and education programmes for buyers of insurance products are often recommended to fill in these ‘*knowledge deficits*.’ Indeed, many recommendations have been offered by both academics and industry to improve financial education in general.^3^ The central motive of this paper, is thus, to re-examine the belief that those who are educated are more likely to buy insurance. We emphasize that there are more important determinants of insurance buying in comparison with insurance literacy. We first stress, through our data from households in Karnataka, that insurance buying behaviour does not significantly differ between those who lack education and those who are sufficiently educated. We argue that education is not a strong determinant of insurance buying, contrary to what such recommendations implicitly assume. Simply put, if education does not significantly improve insurance buying, then the benefits from monetary resources and non-monetary efforts devoted to consumer education may be inconsequential.

We recognize that in some cases, research has demonstrated a negative relationship between health insurance literacy and the likelihood of delayed or foregone care owing to cost for both preventive and non-preventive care.^4^ However, in most cases, not only is financial illiteracy the norm, but those who are financially literate do not show significantly different insurance buying behaviour.^5^

We offer the insight that individuals who have been in a previous situation of losses (where they could easily fathom the benefits from being insured) and who borrowed from usurious sources to meet medical expenses are significantly more likely to purchase insurance regardless of whether or not they are educated. In rural and semi-urban areas, moneylenders and pawnbrokers (who grant credit at exorbitant interest rates) play an essential role during a health crisis. The repayment obligation of high-cost credit would also influence enrolment decisions; thus, the households borrowing from usurious sources are more likely to enrol in micro health insurance (MHI).

The Indian public health system has not yet caught up with the demand of the population of over a billion because of financial (weak tax compliance, and ineffective tax collection machinery) and human resource constraints. Health insurance in India is also under-developed – it is characterized by low levels of government healthcare expenditure (1.18% of gross domestic product) and high out-of-pocket expense (OOPE) that approximately amount to 60.6% of total health expenditure.6 The households in the informal sector fall below the poverty line during illness due to wage loss, catastrophic medical expenses, and repeated medical treatment.^7^ Thus, iatrogenic poverty (defined as medical illness-induced poverty) often leads to further impoverishment of the already poor households when they resort to financing out of savings, borrowing from informal sources, sale of productive assets, paying from current budget by reducing consumption, substituting or increasing labour supply, or reallocation of resources within the household.^8,9^ One-fourth of hospitalized Indians fall below the poverty line after a medical treatment, while more than two-fifths of inpatients borrow or sell assets to meet the treatment cost.^10^ Among these ex-post strategies, informal exploitative credit from money lenders or pawnbrokers, or (sometimes) even microfinance institutions (MFIs) has negative consequences on current financial health and future economic status of households.^11^ Therefore, the Ayushman Bharat Yojana (National Health Protection Scheme), an ambitious (and so far, the largest) social health insurance programme in the world, was launched in 2018 to provide a coverage of INR 0.5 million (1 USD = approximately INR 71 as on October 2019) for over 10 crore poor families.

Before this scheme, several non-government organizations or MFIs offered MHI as an extension of existing micro-credit activities. However, few studies question the financial viability of the schemes owing to a small risk pool, problem of information asymmetry, and excessive reliance on subsidies or external grants.^12-16^ Poor penetration has been identified as one of the prominent reasons for the failure of MHI, a matter of great concern for low- and middle-income countries.^14,17-19^ Low uptake of microinsurance has been observed in African countries.^16,20^ Hence the success of these schemes in achieving universal coverage is debatable if it fails to create value for the poor ensuing lower membership base and limited risk-pooling.^14,18,21,22^ We chose Sampoorna Suraksha Programme (SSP), one of the MHI programmes with largest risk pool in India nested in a broader socio-economic development programme in Karnataka, to understand the determinants of enrolment.

### Literature Review

Enrolment is influenced by hospitalizations (often a proxy for health status), perceived self-health status and chronic illness in the household.^23^ Another study demonstrated that the experience of chronic illnesses in households, education, age, and gender of the head of the households are associated with variation in enrolment.^24,25^ The households having high ratio of ill members and those reporting chronic illness enrol in MHI.^25-27^ Most of these studies however, look at how insurance buying behaviour is *associated*with such socio-demographic variables. We try to go a step further and attempt to establish *causality.*More specifically, for example, the logit regressions in the literature *assume* a well-defined direction of causality from health condition to insurance. In general, it must be emphasized that access to insurance may also *lead to*better well-being in the long run. Such mutual feedback effects between (2 or more) variables of interest should be accounted for in any refined statistical analysis. Therefore, we use a robust three-stage least squares (3SLS) technique (details explained later), instead of unidirectional logit models to bring in a channel of *causality* to the existing research. In a sense, therefore, our contribution can also be seen as methodological. Thus, households that are exposed to higher risk of illness requiring hospitalization or those with higher health expenditure can be expected to enrol in MHI (through that very channel of causality).

There is a direct benefit of understanding causality over association: the interplay of so many variables could make the direction of any association look non-specific – a problem that causality directly addresses. Indeed, prior research findings that have aimed at understanding how enrolment is *associated*with other variables, have arrived at diverse (and mixed) conclusions, to which we turn now.

The households having ill members demand health insurance,^27^ pay more to participate in insurance scheme,^28^ and are more likely to renew the policy.^29^ Individuals with worse health status enrol more than those with better health status.^30^

Health expenditure imposes a burden on the income of the household, and thus may positively influence enrolment.^31-33^ A substantial uptake in health insurance because of escalated healthcare costs has been documented.^34^ Another study highlights the role of current and future health expenditure, and the perception of future healthcare risks, in health insurance purchase decisions.^35^ Clearly, the demand for insurance should include an absolute reduction of hardship financing,^36^ and enrolment is greatly influenced by the desire to reduce this risk of hardship financing. Dror and Firth^37^ argue that individuals do incur very high expenses. The deficit between insurance cover and medical expenses is often financed by usurious credit. So far, adverse selection and its impact on healthcare financing and sustainability have been the focus of earlier studies. Our study goes one step further in demonstrating that in addition to illness, associated usurious borrowing determines enrolment.

Now we explicitly discuss the mixed results on the relation between education and enrolment, that have been highlighted in the literature. Income, which could ease some of the hardship financing discussed above, is often directly linked to education. Clearly, an educated person can be expected to have a higher income and report a positive association between these variables and enrolment.^29,38^ This positive association between income (a proxy for affordability) and health insurance purchase is documented by many studies conducted in different countries.^39^ On the other hand, few studies failed to observe the influence of income in shaping enrolment decisions.^40,41^

Many studies document a positive association between education and risk aversion and hence, higher demand for insurance.^25,42^ Individuals with higher levels of education engage in preventive behaviour and appreciate the benefits of insurance as a protective tool, and hence there is a direct relation between education and demand for health insurance.^43,44^ However, it has been established that a negative association between education and enrolment exists – less educated heads of households are more likely to enrol compared to highly educated heads.^27^ The logic is that less educated agents, on an average, engage in worse healthcare practices (in comparison to those who are educated) and therefore feel a greater need to remain insured.

In a nutshell, therefore, the assumptions of established theories on demand for insurance explaining the role of attitude to risk (Friedman and Savage vs. Kahneman and Tversky), expected utility (von-Neumann and Morgenstern) and moral hazard^45^ may not directly hold in large informal economies such as India. The validity of many axioms could be undermined in the presence of group consensus and collective good,^46^ informal mutual insurance,^47^ low awareness and misinterpretation of information,^48^ difficulty in enforcing contracts,^49^ preference for high-frequency events involving uncertain cost over predictable and low cost events and high variance of OOPEs.^41,50,51^ Refuting the relevance of conventional demand theories for the violation of the underlying assumptions in the informal sector,^37^ calls for a new approach that states that social capital (group affiliation and reciprocity), imperfect market conditions and the perception that health insurance improves community welfare determine enrollment.

In the informal sector, gaining access to unaffordable healthcare services during illness is highly valuable, and thus, the health insurance preference of individuals is greatly influenced by current health, past health behaviour, and health investments. The enrolment models developed by Ito and Kono^25^ and Bonan et al^52^ use household and individual characteristics as a proxy for subjective apprehension and risk behaviour. Outreville^53^ groups the factors determining demand for life insurance under economic, demographic, socio-cultural and institutional categories. Akin to a study by Mahmood et al,^26^ we adopted this framework by incorporating economic factors (income, types of borrowing for medical needs), social factors (education) and demographic factors (illness experience as a proxy for health status), but excluding structural factors such as non-government organization membership given that self-help group (SHG) membership is a prerequisite for buying MHI policy.

## Methods

### Study Context

The SSP was started in 2004 by SKDRDP (Sri Kshetra Dharmasthala Rural Development Project) to provide financial assistance to meet the unexpected medical expenses to the stakeholders and their family, to facilitate access to the best hospitals and to provide medical facilities at lower costs. This voluntary membership-based bundled scheme is offered to SHGs and their family members in the age bracket from 3 months to 80 years. Enrolment of members takes place in February of every year. Sampoorna Suraksha provides medical benefits (health treatment) and exclusive benefits (delivery allowances, death consolation, and domiciliary treatment). The sum assured per member per year is INR 10 000. The scheme offers a family floater cover for 7 members up to INR 70 000, depending on the medical condition and hospital bills. The insured members could get the medical treatment in any of the 110-network hospitals with or without referral from another doctor. In 2010-2011, 1 660 185 members from 420 302 families joined SSP, and INR 364 085 225 was mobilized as premium in 2011-2012. A total of INR 45 5493 625 was given as claim benefits to 133 962 individuals in 2010-2011.

### Study Design

This cross-sectional descriptive study was designed to collect quantitative data using survey methodology in the first half of the year 2011. We are primarily interested in the factors that motivate households to join MHI. We remain open to the possibility that the demand (for insurance) determinants of newly joined households and those of existing insured households need not be the same. The factors that determine enrolment are past illness experience and financial consequences of illness such as borrowing. We also controlled for the monthly income of the family; marital status, age, education and occupation of the head of the households; area of residence for descriptive analysis. Of these, 2 control factors were noteworthy; education (understanding of insurance) and income (affordability of the premium) of the households. Borrowing from high-cost (usurious such as money lenders, pawnbrokers) and low-cost sources (non-usurious such as friends, relatives, neighbours) were related to education level of the head of the households. If the head is more educated, she can be expected to avoid usurious credit. We present our findings using 3SLS regression.^54^ We looked into loans from money lenders/pawnbrokers/MFIs as informal usurious sources of credit. Although MFI is a formal source of finance in India, the credit should be used for productive purposes rather than for consumption smoothing. Since the use of MFI credit for health expenses does not generate income that can be used to pay back the debt, we considered MFI credit for medical care as a source that jeopardizes future household consumption with negative consequences. Thus, it was clubbed with loan from money lenders and pawnbrokers. We coded “0” in the model if credit was taken from non-usurious sources such as neighbours, friends, community members, or relatives.

The data on SSP membership, illness episodes and subsequent costs of treatment in the previous year of the study, types of borrowing during health shocks, cost of treatment and socio-economic characteristics (age, gender, occupation, education, monthly income, marital status, and area of residence) was collected. The questionnaire was piloted and checked for content validity and reliability by using test-retest method. As the target population size was 892 740 households in 2011-2012 (SSP households were 420 302 that included insured and newly insured), 385 was considered as desirable sample size according to the method of binding frontiers.^55^ A multi-stage cluster design with random selection procedures was adopted to select households for the study. In the first stage, 3 districts where SSP was being implemented were selected, and later, 10 *taluks*(administrative regions) from these districts were selected based on literacy index. In the third stage, 18 *valayas*(divisions in each taluk) were chosen from these *taluks,*and later, 84 *karyakshetras* (villages) were randomly selected from the list given by the project office. In the next stage, using the list of households in each *karyakshetra*, 782 households were selected using systematic sampling method. In the sample, 416 were renewed insured, and 366 were newly insured.

## Results

### Socio-Economic Profile of Households

Predominantly, men were found to be heading the households in both groups (newly insured 84.7%; renewed 83.4%) (*P* = .624). The mean age of household head in newly insured households was 48 (SD 10) years, and that of renewed was 47 years (SD 11) (*P* = .150). The mean distance to hospitals for renewed households was 2.3 km and for newly insured 2.8 km (*P* < .05). Each type of household had 4 members on an average (*P* > .05). The monthly income of renewed insured was INR 8773 (SD INR 7076), and newly insured was INR 9738 (SD INR 9609) (*P* = .150). The occupation of most heads of the households in renewed insured and newly insured group was daily labour (Table 1).

**Table 1:** Basic Characteristics of Households

	**Renewed Insured** **(n = 416)**	**Newly Insured** **(n = 366)**	**Test Value**
Education of head of the household (%)			4.22
Illiterate	23.1	26.5	
Primary	43.5	42.1	
Secondary and above	33.4	31.4	
Occupation of head of the household (%)			10.01
Waged labourer	56.5	60.4	
Home maker/unable to work	6.5	4.9	
Self-employment	9.9	5.5	
Formal sector employment	3.1	5.7	
Unemployed	12.3	12.8	
Salaried (informal sector)	8.4	6.8	
Agriculture	3.4	3.8	
Area of residence			14.88^*^
Rural	7.2	14.2	
Urban	40.6	30.6	
Semi Urban	52.2	55.2	

^*^P < .05.

### Incidence of Illness and Health Financing

Nearly 38% of renewed households reported illness episodes, whereas 32.5% of newly insured had incidence of illness in the preceding year of joining SSP (*P* = .09). A larger percentage of households reported chronic illness (54.1% in renewed insured and 45.3% in newly insured), followed by acute illness (43.3% in renewed insured and 48.7% in newly insured) (*P* = .17). In renewed insured group, majority of ill persons in renewed group got inpatient treatment (89.9%), incurred OOPEs of an average of INR 14 816 (SD INR 33 693), and 57.2% of households borrowed with an average borrowing of INR 7505 (SD INR 25 214) to meet the cost of treatment. In comparison with renewed insured, lower proportion of newly insured households had inpatient treatment (70.1%) (*P* = .00), higher OOPE of INR 17 341 (SD INR 36 259) (*P* = .00), and 79.5% borrowed (*P* = .00) with an average of INR 16 495 (SD INR 34 583) (*P* = .00). The mean indirect cost was INR 961 for renewed and INR 1264 for newly insured households (*P* = .56). Of the borrowing sources, usurious credit was used by a higher proportion of newly insured (39.1%) compared to renewed insured (21.2%) (*P* = .01). The amount borrowed was significantly higher for formal sources (average of INR 19 198 and SD INR 25 635) than informal sources (average of INR 14 822 and SD INR 38 912) (*P* = .00).

### Results of the Regression Analysis

In Table 2, we look at the determinants and correlates of non-usurious borrowing. From a linear probability model reported in column (1), we immediately see that those who engage in usurious borrowing are about 62% less likely to engage in non-usurious borrowing (the knowledge of this figure will help us refine our estimates in the regressions that follow). From column (2), we learn that those who are illiterate are not any less likely to go for non-usurious borrowing than those who are literate. Column (3) reports an interesting finding that those who have primary education are less likely to go in for non-usurious borrowing. However, the result in column (4) sums up the story – those with secondary education are more likely to go for non-usurious borrowing.

**Table 2:** Determinants of Non-usurious Borrowing

**Non-Usurious**	**(1)**	**(2)**	**(3)**	**(4)**
Usurious borrowing	-0.62*** (0.02)			
Illiterate		-0.015 (0.03)		
Primary education			-0.06** (0.03)	
Secondary education				0.07** (0.03)
Constant	0.66*** (0.01)	0.55*** (0.02)	0.58*** (0.02)	0.53*** (0.02)

*, **, and *** denote significance levels of 10%, 5% and 1% respectively. Robust standard errors in parentheses.

Now we look for the determinants and the correlates of usurious borrowing. From Table 3, we learn that education level/literacy is not a significant determinant of usurious borrowing behaviour. This is surprising, but we know that during a health crisis, instant payment is to be made primarily in the case of emergency treatment, and the households will be forced to make a choice regardless of their knowledge of demerits of usurious credit.

**Table 3:** Determinants of Usurious Borrowing

**Usurious**	**(1)**	**(2)**	**(3)**
Illiterate	-0.04 (0.02)		
Primary education		0.01 (0.02)	
Secondary education			0.01 (0.02)
Constant	0.17*** (0.01)	0.16*** (0.01)	0.58*** (0.16)

*, **, and *** denote significance levels of 10%, 5% and 1% respectively. Robust standard errors in parentheses.

Since we are ultimately interested in studying the determinants of whether a family chooses to be newly insured, we borrow from the previous regressions (the result that the role of education is limited concerning such decisions) in the combined sets of estimation that follow. In Table 4, we look at the regression estimates of a linear probability model where the left-hand side is the probability that a household will be newly insured (the standard interpretation of a dummy variable on the left-hand-side). In column (1), we immediately see that those who are already insured are 50% less likely to take up new insurance. This means that new insurance buyers are mostly those who do not already have insurance. In column (2), we introduce an additional control with a dummy for whether a family (when deciding to buy insurance), has already seen instances of illness in the recent past. New insurance buyers are those who do not already have insurance and have seen illness in recent past. We want to exploit our knowledge of this simple fact to refine our estimates. In columns (3) and (4), we add controls for education levels and see that our results are robust. Finally, in column (5), we introduce a control for family (monthly) income, since those who have secondary education are often associated with higher earning capacities (and in turn, those with higher incomes can afford secondary education, let alone insurance). We see that our finding that education is not as strong a determinant of insurance buying as much as experience (of illness) is, stands. Because of the potential reverse causality issue between the income and education variables, we look at the estimates from a 3SLS regression in column (6). The results are similar when we replace non-usurious borrowing by usurious borrowing (although the coefficient of the latter is more significant).

**Table 4:** Determinants of the Decision to be Newly Insured

**Newly Insured**	**(1) OLS**	**(2) OLS**	**(3) OLS**	**(4) OLS**	**(5) OLS**	**(6) 3SLS**
Already insured	-0.51*** (0.01)	-0.51*** (0.01)	-0.51*** (0.01)	-0.51*** (0.01)	-0.51*** (0.01)	-0.51*** (0.02)
Illness		0.06*** (0.02)	0.06*** (0.02)	0.06*** (0.02)	0.06*** (0.02)	0.07*** (0.02)
Primary education			0.01 (0.02)			
Secondary education				-0.03 (0.02)		
Income					-8.01e-07 (1.20e-06)	
Non-usurious borrowing						-0.05** (0.02)
Constant	0.50*** (0.01)	0.48*** (0.01)	0.48*** (0.02)	0.49*** (0.02)	0.48*** (0.02)	0.51*** (0.02)
R-Squared	0.26	0.27	0.27	0.27	0.27	0.27

*, **, and *** denote significance levels of 10%, 5% and 1% respectively. Robust standard errors in parentheses.

So far, we have provided naïve regressions with stringent covariance restrictions. We now look at estimates from reduced-form models, simultaneously determined using 3SLS.

In Table 5, we borrow the results in columns (1) and (2) of Table 4. We show the simultaneous estimation of the following 2 equations that capture a mutual feedback effect.

**Table 5:** Determinants of the Decision to Be Newly Insured

**Dependent Variable**	**Newly Insured 3SLS (1)**	**Non-usurious Borrowing 3SLS (2)**	**Newly Insured** **3SLS (3)**	**Non-usurious Borrowing 3SLS (4)**	**Newly Insured** **3SLS (5)**	**Non-usurious Borrowing 3SLS (6)**
Already insured	-0.50*** (0.02)		-0.50*** (0.02)		-0.50*** (0.02)	
Illness	0.06** (0.02)		0.06** (0.02)		0.06** (0.03)	
Usurious borrowing	0.21*** (0.03)		0.22*** (0.03)		0.22*** (0.03)	
Newly insured		-0.23*** (0.06)		-0.23*** (0.06)		-0.21*** (0.05)
Income			-5.94e-07 (1.45e-06)	7.00e-07 (1.85e-06)	-8.41e-07 (1.45e-06)	1.56e-07 (1.84e-06)
Secondary education			-0.04* (0.02)	0.06** (0.03)	-0.04* (0.02)	0.05* (0.03)
Constant	0.44*** (0.01)	0.63*** (0.02)	0.46*** (0.02)	0.60*** (0.03)	0.39*** (0.05)	0.43*** (0.06)
Chi-square	463.32***	14.78***	467.81***	21.40***	480.32***	42.43***

*, **, and *** denote significance levels of 10%, 5% and 1% respectively. Robust standard errors in parentheses.

*Newly insured = α*_0_ *+α*_1_ *insured + α*_2_ *usurious borrowing + α*_3_ *ill +* ***Xβ*** *+ u*(1)

*non-usurious borrowing = μ*_0_ *+ μ*_1_ *newly insured +* ***Xγ*** *+ v*(2)

Where, *newly insured*, *insured, usurious borrowing, non-usurious borrowing*, and *ill*are dummy variables that capture whether a decision to be newly insured was taken, whether or not the household was already insured, whether or not a household engaged in usurious borrowing, whether or not a household engaged in non-usurious borrowing, and whether or not a household saw illness in the family in a recent past. ***X ***is a vector of covariates (including education level) associated with the coefficient vectors *β* and *γ*in regressions (1) and (2). *u* and *v* are stochastic error terms. Regression (2) above tells us the likelihood with which non-usurious borrowing will decline when a household chooses to be newly insured. This reduction in non-usurious borrowing may translate to higher levels of usurious borrowing (since there is a negative relation between usurious and non-usurious borrowing). We finally see if this change in usurious borrowing can further influence the decision to be newly insured in regression (1). The coefficients obtained from the simultaneous estimation of (1) and (2) will help us explicitly understand this feedback loop, and therefore provide us with an accurate understanding of the relative effects of education and experience.

Even after accounting for the mutual feedback effect, we see in columns (1) and (2) that a household is more likely to buy new insurance if it is not already insured and if it has experienced illness in the recent past. It seems that an experience of illness in a household is costly when it is not insured. The household additionally, incurs psychological costs of ex-post regret when not insured at the time of experiencing an ailment or suffering. So, who all go for the usurious borrowing? In columns (3) and (4), we report regression estimates for the same equations (1) and (2), with additional controls for income and education. We see that our central findings stand. We conclude that experience (of witnessing a prior suffering/illness) matters in the decision to buy new insurance and not education. With further robustness checks as controls for potential determinants including the social activities and involvements with families in columns (5) and (6), we see that our central results continue to hold. The strength of our regression specification is in this robustness in its predictive capacity.

## Discussion

The analysis presented above depicts the relationship between enrolment in MHI and usurious borrowing owing to the incidence of illness in the household. This finding has not been frequently reported in the published literature although there is enough evidence on the role of education and income in shaping enrolment decisions in MHI. When controlling for education and income, we find newly insured to join SSP to avoid high OOPE and the consequent usurious borrowing. The inadequacy of informal risk-sharing arrangements in the absence of MHI forces people to borrow from usurious sources for even frequent uncertain medical costs that have negative consequences on current and future financial status of the household.^12^ Despite being a member of SHGs, newly insured did not participate in SSP when it was offered voluntarily. After the household experienced illness and its drastic financial consequences measured by high OOPE and hardship financing in the form of usurious borrowing, newly insured families joined the risk pool of SSP. In support of this finding,^26,27,31-34^ research confirms a positive relationship between health expenditure risk aversion and participation in MHI. These households become risk-averse to avert negative financial consequences of future health shocks. Newly insured rely more on informal usurious sources of finance during health shocks and not non-usurious sources (Tables 4 and 5). This may be because, as shown in the results section, renewed insured claimed from SSP reduced OOPE, whereas newly insured had greater OOPE necessitating borrowing from multiple sources, including usurious and non-usurious credit with severe financial consequences on the future well-being of the household. We cannot deny that renewed insured too used usurious credit because of insufficient sum insured, exclusion of outpatient treatment and indirect costs; however, the magnitude was less compared to newly insured. When the successful claim stories unfold among the SHG members, uninsured would be more inclined to enrol in SSP. Thus, for the poor households, income or affordability may not be an obstacle to enrol; even education may not be a hindrance. The experience of illness and its repercussions on the household giving rise to post-regret has a significant influence on the decision to enrol in MHI.

We add to the ongoing debate whether informal networks create obstacles to the enrolment in formal insurance from our study findings that informal usurious network motivates households to be a part of formal health insurance when the poor households attempt to extricate from the clutches of usurious lenders. As corroborated by Dror and Firth,^37^ in our study uninsured households resorting to hardship financing cannot transfer either healthcare costs or cost of borrowing to others, they would enrol in MHI to reduce the variance of high OOPE and the consequent usurious borrowing and its associated adverse financial implications. Nyman^45^ advocates health insurance as welfare promoting measures in developing countries. Similarly, if usurious borrowers enrol, MHI would foster welfare by reducing the reliance on moneylenders during future shocks. Moreover, the credit-constrained households are less likely to purchase private insurance schemes^56^; hence it is imperative for the countries facing iatrogenic poverty and borrowing heavily from usurious sources during illness to contemplate launching government-sponsored social health insurance scheme and promote MHI.

Nevertheless, inclusion of households having a high level of usurious debt and risk of illness would increase both the high-risk households in the risk pool and the consequent high claims ratio in SSP. Despite insisting on household as the unit of enrolment, features such as lack of waiting period, inclusion of pre-existing diseases, and upper age for enrolment being 85 years increases the scope for adverse selection. Hence, financial viability of SSP depends on the willingness of for-profit insurance companies to continue their partnership with the scheme despite high claims ratio.

Our study negates an association between education (information access) and income (affordability of premium) and enrolment in MHI in contrast to previous studies that had established positive relationship between education and enrolment.^24,25,42,44,57^ It was also observed that if the head of the household has completed secondary education or higher, the family’s tendency to borrow from low-interest usurious sources such as relatives, friends, and neighbours would be more. Given that SSP mainly caters to the needs of rural households in which heads of households usually have less education, the finding of the study is not surprising. Firstly, while education is taken as a demand-side variable, it may not reflect the understanding of insurance value proposition and desirable credit behaviour.^24^ Thus, financial literacy instead of education (measured in terms of years of schooling) would create a better understanding of value of insurance and flaws of usurious credit. Secondly, education may not play a role in health crisis when high level of OOPE is to be made in less time. Thus, for effective risk management, MHI managers must engage in user-friendly marketing activities that enhance financial literacy of poor.

The study finding that income was not a determinant of enrolment is supported by Polonsky et al^40^ and Panda et al^41^ but is contradicted by other studies.^4,57^ As Dror et al^24^ argue, affordability of premium is not the same as income; ready availability of liquid cash (after harvest season or payment in instalments) during enrolment period determines affordability. SSP targets below poverty line families; however, relatively higher-income families in this study are still poor when we consider the definition of the income quintiles given by Planning Commission on all-India basis. Besides, SSP collects premiums in February every year, and some households may not be able to pay a lumpsum owing to seasonality of cash flows in informal and rural areas, even if they have income to pay the premium.

The findings of the study can be applied to other contexts characterized by a sizeable informal economy where conventional assumptions of theories of insurance demand are invalid. The scheme administrators aiming to increase demand for MHI should encourage the formation of bottom-up community-based organizations that promote solidarity, reciprocity, mutual trust, and informal non-usurious risk-sharing arrangements. Instead of enrolling in MHI after experiencing illness and undergoing financial hardships owing to usurious borrowing, awareness, and perception of health insurance as a risk coping strategy and welfare-enhancing mechanism should be stressed in policy propaganda. The study findings are not generalizable; however, it applies to similar MHI schemes initiated by MFIs elsewhere in Karnataka.

## Conclusion

In the absence of appropriate and adequate health financing mechanisms to pay for the high cost of treatment, the informal credit market in rural India flourishes, pushing the poor households into debt trap. MHI is a preferred alternative to informal usurious financing, as evidenced by the enrolment of newly insured households. Yet, newly joined insured reported past illness, incurred huge OOPEs and owed to usurious lenders. This finding suggests the prevalence of adverse selection in SSP; having high-risk individuals in the risk pool may be acceptable from social welfare perspective but questionable from the viability view point. Our finding stresses the considerable responsibility of the scheme administrators to scrutinize the risk profile of prospective clients to safeguard financial sustainability.

## Acknowledgements

We thank Gatton College of Business and Economics, University of Kentucky for awarding Kalam Travel Grant to present an earlier version of this paper at the Kalam Research Conference (organized by Gatton College of Business and) on September 23, 2016 at the University of Kentucky, Lexington, USA.

## Ethical issues

The ethical approval was obtained from the executive director of SSP, SKDRDP, Karnataka.

## Competing interests

Authors declare that they have no competing interests.

## Authors’ contributions

BS: Conceptualization of the study, literature review, data collection, analysis and discussion. SB: Background, analysis of the data and discussion. This research question was decided by SB.

## Authors’ affiliations

^1^Manipal Institute of Management, Centre for Advanced Research in Financial Inclusion, Manipal Academy of Higher Education, Manipal, Karnataka, India. ^2^University of Melbourne (Australia India Institute), Melbourne, VIC, Australia. ^3^Queensland University of Technology (BEST Centre), Brisbane, QLD, Australia.

## Key Messages

**Implications for policy makers**

- Adverse selection and the consequential financial non-sustainability must be curtailed through a scrutiny of the risk-profile of prospective clients.
- Scheme administrators could collect data on illness and borrowing habits that concern the social capital of rural communities.
- In addition to the compulsory enrolment of all family members, a waiting period of one month could be enforced.
- Instead of risk-rating on the part of the community, one could adopt the sliding scale methodology to determine premiums (and consequently, charge higher premiums for high-risk individuals).

**Implications for public**

For impoverished households, income and education may not be obstacles to enrolment. The experience of illness and its repercussions on the household (giving rise to ex-post regret for not being insured), however, has a significant influence on the decision to (eventually) enrol in micro health insurance (MHI). While insurance (of renewed policy) claims from the MHI scheme reduced out-of-pocket expenses (OOPEs), those newly insured had a comparably higher OOPE, necessitating higher borrowing from multiple sources including usurious and non-usurious credit. Since usurious loan has severe consequences on the financial well-being of any household, the non-insured joined Sampoorna Suraksha Programme (SSP, which is formally explained later) to mitigate the impact of future (adverse) health shocks.
